# Predicting Geriatric Rehabilitation Stays of ≤4 Weeks After Hip Fracture Surgery: Machine Learning Approach Using Physical Activity and Patient Data

**DOI:** 10.2196/79331

**Published:** 2026-02-23

**Authors:** Sanne M Krakers, Frank J Wouda, Dieuwke van Dartel, Miriam MR Vollenbroek-Hutten, Johannes H Hegeman, Up&Go After a Hip Fracture Group

**Affiliations:** 1 Department of Biomedical Signals and Systems University of Twente Enschede The Netherlands; 2 Department of Trauma Surgery Ziekenhuisgroep Twente Almelo The Netherlands; 3 Reggeborgh Research Fellow ZGT Academy Ziekenhuisgroep Twente Almelo The Netherlands; 4 Board of Directors Medisch Spectrum Twente Enschede The Netherlands; 5 See Acknowledgments

**Keywords:** accelerometer, continuous physical activity monitoring, geriatric rehabilitation, hip fracture, length of stay, machine learning, prediction

## Abstract

**Background:**

In 2022, over 18,000 patients aged ≥70 years were hospitalized in the Netherlands for a hip fracture, with 50% requiring geriatric rehabilitation after surgery. Increasing geriatric rehabilitation patient numbers, staff shortages, and rising pressure on health care budgets make adequate care challenging. To make geriatric rehabilitation more future-proof, a stronger focus on home-based rehabilitation is needed. Early identification of patients likely to be discharged soon enables timely discharge planning and coordination of support at home. Early geriatric rehabilitation discharge planning may help organize home-based rehabilitation more effectively by arranging home care services in advance. This can facilitate smoother transitions toward home and prevent discharge delays, which is important to ensure optimal bed occupancy.

**Objective:**

This study aims to develop machine learning (ML) models to predict a geriatric rehabilitation stay of 4 weeks or less in a skilled nursing home for older patients after hip fracture surgery, using continuously monitored physical activity data from the first week of geriatric rehabilitation and patient characteristics.

**Methods:**

This prospective cohort study (January 2019-August 2024) included 100 patients. Patient characteristics and physical activity data from the MOX1 accelerometer (Maastricht Instruments BV) were collected during the first rehabilitation week. Principal component analysis was used to reduce the physical activity features. ML models were developed using Bayesian hyperparameter optimization and refined if necessary. The performance of the single best-performing configuration per remaining ML model type was evaluated, and the most important features for predicting the length of geriatric rehabilitation stay were identified.

**Results:**

Of the 3 ML models evaluated (support vector machine [SVM], ensemble of decision trees, and neural network), the SVM achieved the highest performance, with 19 out of 20 correct predictions (accuracy=0.95, 95% CI 0.85-1.00; precision=0.91, 95% CI 0.71-1.00; recall=1.00, 95% CI 1.00-1.00; *F*_1_-score=0.95238, 95% CI 0.83-1.00; area under the curve [AUC]=0.97, 95% CI 0.83-1.00). The most important features for predicting the length of geriatric rehabilitation stay across the best-performing ML models included the continuously monitored physical activity data, time in the emergency room, functional ambulation category (FAC) at hospital discharge, age, Katz Index of Independence in Activities of Daily Living–6 (Katz-ADL6) at hospital discharge, Montreal Cognitive Assessment (MoCA), availability of nonprofessional help, surgery type, Charlson Comorbidity Index (CCI), gender, and hemoglobin level at hospital admission.

**Conclusions:**

This study developed several ML models to predict a geriatric rehabilitation stay of ≤4 weeks in a skilled nursing home for older patients after hip fracture surgery. Among these models, the SVM proved to be highly accurate in its predictions with an accuracy of 0.95 (95% CI 0.85-1.00), precision of 0.91 (95% CI 0.71-1.00), recall of 1.00 (95% CI 1.00-1.00), *F*_1_-score of 0.95 (95% CI 0.83-1.00), and AUC of 0.97 (95% CI 0.88-1.00).

## Introduction

In the Netherlands, more than 18,000 patients aged 70 years and older were hospitalized with a hip fracture in 2022 [1]. After hip fracture surgery, more than 50% of the patients are admitted to a geriatric rehabilitation department at a skilled nursing home [2,3]. Geriatric rehabilitation is defined by the European Consensus group as “a multidimensional approach involving diagnostic and therapeutic interventions. Its purpose is to optimize functional capacity, promote activity, and preserve functional reserve and social participation in older people with disabling impairments” [4]. Based on incidence trends and increasing life expectancy, the number of hip fracture patients is expected to double by 2050 [5]. The expectation is that the group of geriatric rehabilitation patients will also increase. However, the number of professionals able to provide care for these patients will not grow at the same pace. In addition, health care budgets are under increasing pressure [6]. Together, these developments make the provision of geriatric rehabilitation care increasingly challenging.

To make geriatric rehabilitation future-proof, a stronger focus on home-based rehabilitation is essential, especially given the shifting vision of Dutch health care that emphasizes treatment closer to home [7]. Achieving this requires the early identification of patients who are likely to be discharged within 4 weeks of geriatric rehabilitation admission. Such early identification enables timely discharge planning and the coordination of necessary support at home. The 4-week cutoff marks the transition between shorter and longer rehabilitation trajectories, as defined by the Dutch Diagnosis Treatment Combinations. Early geriatric rehabilitation discharge planning may help organize home-based rehabilitation more effectively by arranging home care services in advance. This can facilitate smoother transitions toward home and prevent unnecessary discharge delays, which is important for maintaining optimal bed occupancy.

Early geriatric rehabilitation discharge planning requires prediction models to make objective predictions for the length of geriatric rehabilitation stay. By relying on data rather than assumptions, these models enable more accurate discharge planning. Machine learning (ML) is a subset of artificial intelligence that enables computers to learn from data without being explicitly programmed [8-10]. The use of ML for developing length of stay prediction models in various medical fields has shown promising results [8,10-12]. However, to the best of our knowledge, no ML models exist that predict the length of geriatric rehabilitation stay at a skilled nursing home for older patients following hip fracture surgery. In a recent study, we identified factors that influence the length of geriatric rehabilitation stay [13]. However, this study did not aim to predict the exact length of geriatric rehabilitation stay, and its multivariate model explained only 32% of the variance. This suggests that additional factors influencing the length of stay exist that were not included in the study. Since early mobilization and physical activity enhance functional recovery, continuously monitored physical activity using accelerometers during the first week of geriatric rehabilitation might be a relevant factor to include [14-16]. Another important factor could be cognitive functioning, assessed with a cognitive screening test, as patients with cognitive impairments may have difficulty performing physiotherapy exercises [17].

The aim of this study is to develop ML models to predict a geriatric rehabilitation stay of ≤4 weeks in a skilled nursing home for older patients after hip fracture surgery, using continuously monitored physical activity data from the first week of geriatric rehabilitation and patient characteristics.

## Methods

### Study Design

A prospective cohort study was conducted from the first of January 2019 until the first of August 2024. Older patients aged ≥70 years who underwent hip fracture surgery at the Centre for Geriatric Traumatology at Ziekenhuisgroep Twente (ZGT) and rehabilitated at one of the 3 participating skilled nursing homes (TriviumMeulenbeltZorg, ZorgAccent, and Carintreggeland) were included. Exclusion criteria were pathological or periprosthetic fractures, severe cognitive impairment, total hip replacement, plaster allergy, or contact isolation. Patients were enrolled one day after hip fracture surgery or one day before discharge to one of the collaborating skilled nursing homes.

### Data Collection

The primary outcome was the length of geriatric rehabilitation stay at the skilled nursing homes, stratified into 2 lengths of stay groups, including ≤4 and >4 weeks. This division was based on the structure of the Dutch Diagnosis Treatment Combinations, which fund geriatric rehabilitation and classify rehabilitation trajectories into one of 5 length of stay groups (1-2, 2-4, 4-8, 8-13, and 13-17 weeks) [18,19]. The 4-week cutoff corresponds to the transition point between shorter and longer rehabilitation pathways.

For this study, the physical activity of all enrolled patients was continuously monitored during the first week of geriatric rehabilitation. Patient characteristics up to the first week of rehabilitation were collected from a transmural care pathway database. This care pathway was implemented to synchronize the care processes between the hospital and geriatric rehabilitation departments and provide more insight into the rehabilitation process of older patients after hip fracture surgery. Patients with an incomplete data collection (<5 days of physical activity data of the first week of geriatric rehabilitation and/or missing patient characteristics) were excluded from the data analysis.

The physical activity of all enrolled patients was continuously monitored using an MOX1 accelerometer (Maastricht Instruments BV) attached with a custom-made patch 10 cm proximal of the patella on the ipsilateral side of the operated hip. This small, lightweight, waterproof device (35x35x10 mm, 11 g, IPX8) contains a tri-axial accelerometer sensor, which measures raw acceleration data for the X-, Y-, and Z-axes at a sampling frequency of 25 Hz, storing the data directly in its internal memory (1.5 GB) for up to 7 days. Data analysis was performed offline after the raw acceleration data were uploaded to a computer via a USB connection.

The collected patient characteristics (baseline, in-hospital, hospital discharge, and geriatric rehabilitation variables; Textbox 1) were selected based on previous studies demonstrating their value in predicting rehabilitation outcomes or length of stay [20-31].

**Patient characteristics included in this study.**

**Baseline variables**

- Age
- Gender
- Premorbid living situation
- Living alone versus living together
- Availability of nonprofessional help
- Need for climbing stairs
- Prefracture Mobility Score (PFMS)
- Katz Index of Independence in Activities of Daily Living-6 (Katz-ADL6)
- Charlson Comorbidity Index (CCI)
- American Society of Anesthesiologists physical status classification (ASA) score
- Short Nutritional Assessment Questionnaire (SNAQ)

**In-hospital variables**

- Hemoglobin level at hospital admission
- Hip fracture type
- Time spent in the emergency room
- Surgical treatment
- Postoperative weight-bearing protocol
- In-hospital complications

**Hospital discharge variables**

- Functional Ambulation Categories (FAC) at hospital discharge
- Fracture Mobility Score (FMS) at hospital discharge
- Katz-ADL6 at hospital discharge
- Discharge destination (skilled nursing home)
- Length of hospital stay

**Geriatric rehabilitation variables**

- Montreal Cognitive Assessment (MoCA)

### Data Analysis for Accelerometer

The raw acceleration data were analyzed using a MATLAB algorithm (R2024b; The MathWorks, Inc) developed and validated for older hospitalized patients by Van Dijk-Huisman et al [32]. This resulted in the total intensity of physical activity per day by determining the signal magnitude area of the acceleration data; the total time spent sitting or lying, standing, and walking per day; the position classification (sitting or lying, standing, walking) every 4 seconds; as well as the total measurement time per day. The intensity of physical activity reflects the raw accelerometer output and is expressed as counts per day, not as categorical levels of activity. If the position classification changed between 2 consecutive 4-second time steps, it was defined as a transition. For each patient, the following metrics were calculated for each day:

- The intensity of physical activity (counts per hour)
- Time spent sitting or lying (minutes per hour)
- Time spent standing (minutes per hour)
- Time spent walking (minutes per hour)
- Transitions between sitting or lying and standing (number of transitions per hour)
- Transitions between sitting or lying and walking (number of transitions per hour)
- Transitions between standing and walking (number of transitions per hour)

Different features were extracted from each metric, including the overall mean, overall SD, weekend mean, weekend SD, weekday mean, weekday SD, median, IQR, root mean square, variance, minimum value, maximum value, minimum-maximum range, coefficient of variance, skewness, kurtosis, and characteristics of the third-degree polynomial curve describing the shape of the pattern. The characteristics of the third-degree polynomial curve were defined as coefficients a, b, c, and d, which were derived from the third-order polynomial equation: y = ax^3^ + bx^2^ + cx + d. More detailed information about the different continuously monitored physical activity features can be found in Multimedia Appendix 1.

After data analysis, data within each of the 2 length-of-stay groups were randomly divided into a training (80%) and testing (20%) set, which were then combined to ensure class balance.

### Feature Reduction

Principal component (PC) analysis, a common method for feature dimensionality reduction, was applied exclusively to the features extracted from the physical activity data of the training set. As a preprocessing step, the data were standardized to have zero mean and unit variance. Each PC, obtained after applying PC analysis, consists of a weighted combination of the features extracted from the physical activity data. The PCs were sorted in decreasing order of their eigenvalues, indicating how much of the data’s variance is captured by its corresponding PC. The PCs contributing to a cumulative explained variance of 95% were selected as input for the ML models.

Feature selection was also applied to the patient characteristics of the training set. Features that did not occur or exhibited an identical distribution between the 2 lengths of stay groups were excluded, as they provided no discriminative value for the ML models. Feature selection was further guided by prior research [13]. When variables were conceptually linked or measured similar aspects of a patient’s condition, priority was given to the variable that was more commonly used in clinical practice or provided a more direct assessment of functional status. The remaining patient characteristics were incorporated into the ML models along with the selected PCs.

### ML Models

First, the performance of 5 commonly used supervised ML models for classification was considered: support vector machine (SVM), neural network (NN), decision tree, naïve Bayes (NB), and an ensemble of decision trees (EoDT). These models were selected because they represent a diverse set of widely used supervised learning models, allowing for a comprehensive evaluation of which modeling strategy is most suitable for this clinical prediction task. For each model, hyperparameters were optimized using Bayesian optimization with an expected-improvement-plus acquisition function and tenfold cross-validation on the training set. Bayesian optimization was chosen because it enables efficient hyperparameter tuning and allows for the development and comparison of multiple ML models simultaneously.

For the SVM, 4 kernel functions (linear, radial basis function, polynomial, and Gaussian) were evaluated separately, allowing the kernel-related model performance to be systematically compared. For each kernel, Bayesian optimization tuned the remaining hyperparameters. For the NN, decision tree, NB, and EoDT, all model-specific hyperparameters were included in the Bayesian optimization procedure. The optimal hyperparameter values for each ML model were determined by the minimum observed value of the objective function.

Running Bayesian optimization for all models over many iterations can be computationally expensive. Therefore, model performance was evaluated after the first 30 iterations to identify a subset of well-performing models. In this screening phase, multiple configurations of the same model type, for example, the SVM models with different kernels, could be retained if they showed similar performance. The area under the receiver operating characteristic curve was used for initial model comparison, after which the models with the highest area under the curve (AUC) underwent continuous Bayesian optimization for an additional 30 iterations. Subsequently, the final hyperparameters of these ML models were evaluated. If notable deviations were identified, a new ML model was trained with adjusted hyperparameters.

Finally, only the single best-performing configuration per remaining ML model type was evaluated on the test set. Model performance was assessed using the confusion matrix, actual length of stay of the misclassified patients, accuracy score, precision score, recall score, *F*_1_-score, and AUC. CIs were obtained using bootstrap resampling; the test set was resampled with replacements 1000 times while keeping the training set and ML models fixed. The 95% CIs were defined by the 2.5th and 97.5th percentiles. A Shapley Additive Interpretation (SHAP) summary plot based on the test set was used to determine the relationship between the 2 length of stay groups and its main predictors for each of the best-performing ML models [33]. The overall best-performing ML model was retrained and retested using 2 additional dataset configurations, (1) patient characteristics only and (2) physical activity data only, to conduct an ablation study evaluating the contribution of each dataset compared with the combined datasets.

### Ethical Considerations

The Medical Ethical Committee of Twente stated that this study did not require an assessment by the Medical Ethical Committee according to Dutch law. The study was approved by the Institutional Review Board of ZGT (ZGT17-40). All patients gave written informed consent to participate. Study data obtained from the enrolled patients were deidentified and managed in accordance with relevant data protection regulations. Patients did not receive any financial or material compensation for their participation.

## Results

### Patient Characteristics and Physical Activity Data

**Figure 1:**
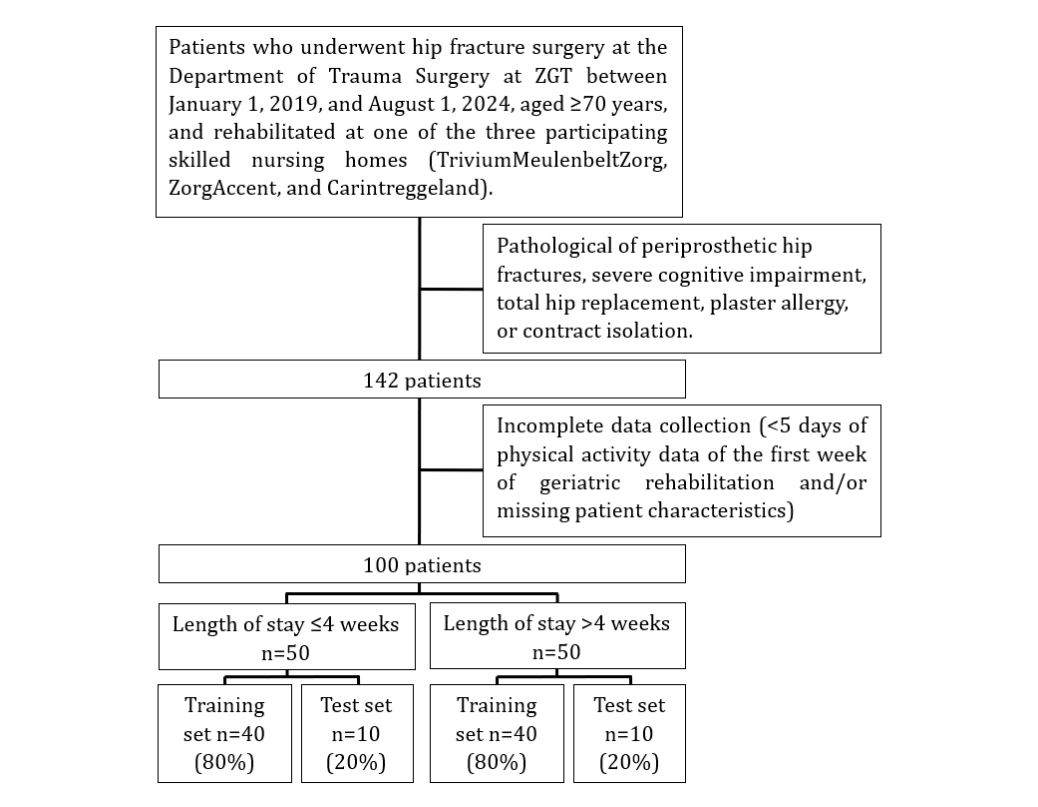
Flowchart patient recruitment.

**Figure 2:**
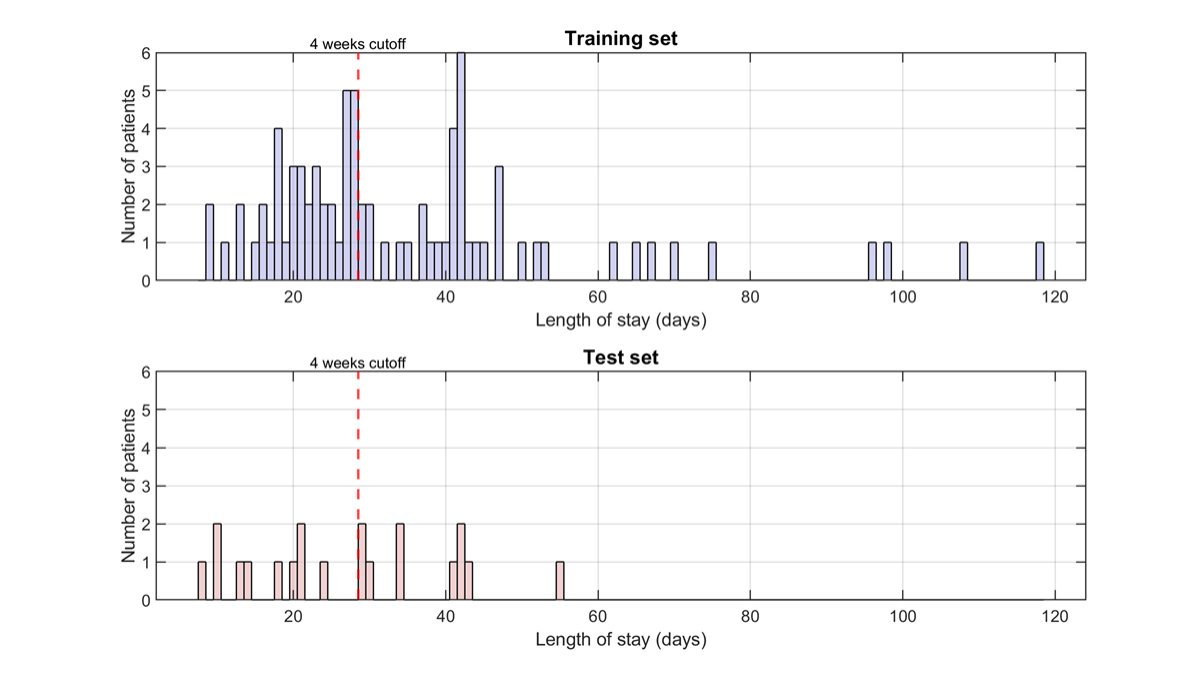
Length of geriatric rehabilitation stay (days): training set vs test set.

A total of 142 older patients were included in this study (Figure 1). Of these, 42 were excluded due to incomplete data (<5 days of physical activity data of the first week of geriatric rehabilitation and/or missing patient characteristics). The missing physical activity data were mainly caused by sensor-related issues, such as battery depletion and removal of the device for charging and data upload. This resulted in a final sample of 100 patients. Patients were divided into ≤4 weeks (n=50) and >4 weeks (n=50) length of geriatric rehabilitation stay and subsequently split into a training and test set. The distribution of the length of geriatric rehabilitation stay of both sets can be seen in Figure 2. Patient characteristics of the training and test set are shown in Table 1. The mean age in the ≤4 weeks group was 84.5 (SD 5.9) years, while the mean age in the >4 weeks group was 82.8 (SD 6.9) years. The median Montreal Cognitive Assessment (MoCA) score was 21.5 (IQR 18-24) and 19.5 (IQR 17-22.5), respectively. The patient characteristics of the test set showed similar results.

**Figure 3:**
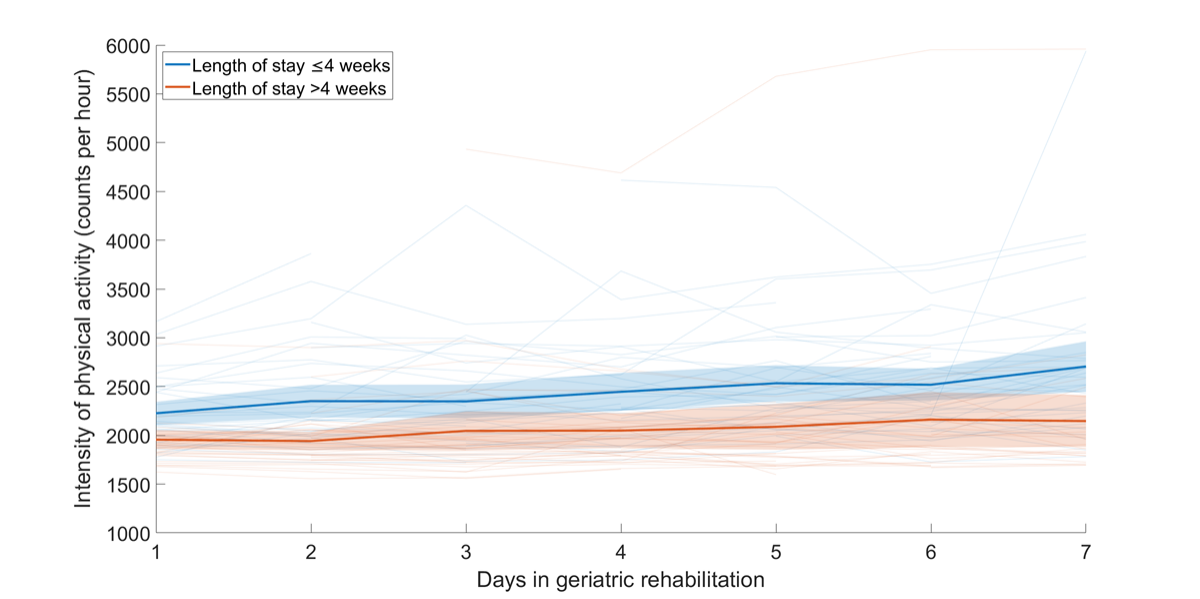
Intensity of physical activity with individual trajectories of the training set and mean physical activity intensity with 95% CI.

Individual patient trajectories of intensity of physical activity during the first 7 days of geriatric rehabilitation are displayed, along with the mean intensity for patients with a length of stay of 4 weeks or less and for those with more than 4 weeks, including 95% CI (Figure 3).

**Table 1:** Patient characteristics training and test set.

Patient characteristics training and test set			Training set Length of geriatric rehabilitation stay			Test set Length of geriatric rehabilitation stay	
			≤4 weeks (n=40)	>4 weeks (n=40)	≤4 weeks (n=10)		>4 weeks (n=10)
**Baseline variables**							
	Age (years)^a^, mean (SD)		84.5 (5.9)	82.8 (6.9)	82.8 (6.0)		82.2 (6.9)
	Female (gender)^a^, n (%)		27 (68)	33 (83)	7 (70)		8 (80)
	**Premorbid living situation^a^, n (%)**						
		Independent without home care services	30 (75)	26 (65)	8 (80)		9 (90)
		Independent with home care services	9 (23)	14 (35)	1 (10)		1 (10)
		Residential home	1 (3)	0 (0)	1 (10)		0 (0)
	Living together^a^, n (%)		12 (30)	14 (35)	2 (20)		7 (70)
	Availability nonprofessional help^a^, n (%)		31 (78)	38 (95)	6 (60)		7 (70)
	Need for climbing stairs^a^, n (%)		11 (28)	7 (18)	4 (40)		3 (30)
	**PFMS^a,b^, n (%)**						
		1-Freely mobile without aids	20 (50)	11 (28)	5 (50)		4 (40)
		2-Mobile outdoors with one aid	4 (10)	1 (3)	1 (10)		2 (20)
		3-Mobile outdoors with 2 aids or frame	16 (40)	27 (68)	4 (40)		4 (40)
		4-Some indoor mobility but never goes outside without help	0 (0)	1 (3)	0 (0)		0 (0)
		5-No functional mobility	0 (0)	0 (0)	0 (0)		0 (0)
	Premorbid Katz-ADL6^a,^^c^, median (IQR)		0 (0-0)	0 (0-1)	0 (0-0)		0 (0-1)
	CCI^a,d^, median (IQR)		1 (0-2)	1 (0-2)	0 (0-1)		1 (0-3)
	**ASA**^e^**score**^a^, n (%)						
		ASA 1	0 (0)	2 (5)	1 (10)		1 (10)
		ASA 2	15 (38)	10 (25)	4 (40)		1 (10)
		ASA 3	23 (58)	22 (55)	3 (30)		6 (60)
		ASA 4	2 (5)	6 (15)	2 (20)		2 (20)
		ASA 5	0 (0)	0 (0)	0 (0)		0 (0)
	SNAQ^a,f^, median (IQR)		0 (0-0)	0 (0-0)	0 (0-0)		0 (0-0)
**In-hospital variables**							
	Hemoglobin level at hospital admission^a^, median (IQR)		7.9 (7.6-8.7)	7.8 (7.5-8.6)	7.9 (7.5-8.1)		7.9 (6.0-8.3)
	**Hip fracture type,** **n** **(%)**						
		Femoral neck	24 (60)	17 (43)	5 (50)		3 (30)
		Subtrochanteric femur	16 (40)	23 (58)	5 (50)		7 (70)
	Time in the emergency room in minutes^a^, median (IQR)		133.5 (103.5-168.0)	133.0 (106.5-161.0)	104.5 (82.0-155.0)		158.5 (124.0-184.0)
	**Surgical treatment^a^, n (%)**						
		Dynamic hip screw	3 (8)	3 (8)	2 (20)		1 (10)
		Intramedullary nail	15 (38)	25 (63)	5 (50)		6 (60)
		Hemiarthroplasty	22 (55)	12 (30)	3 (30)		3 (30)
	**Postoperative weight-bearing protocol^a^, n (%)**						
		Full weight-bearing	40 (100)	36 (90)	10 (100)		9 (90)
		Partial or non–weight-bearing	0 (0)	4 (10)	0 (0)		1 (10)
	**In-hospital complications,** **n** **(%)**						
		Anemia^a^	6 (15)	7 (18)	1 (10)		3 (30)
		Heart failure^a^	1 (3)	2 (5)	0 (0)		1 (10)
		Pressure ulcers^a^	3 (8)	2 (5)	0 (0)		0 (0)
		Delirium^a^	1 (3)	5 (13)	0 (0)		0 (0)
		Pulmonary embolism^a^	0 (0)	1 (3)	0 (0)		0 (0)
		Kidney failure^a^	0 (0)	1 (3)	0 (0)		0 (0)
		Pneumonia^a^	3 (8)	4 (10)	0 (0)		0 (0)
		Urinary tract infection^a^	2 (5)	1 (3)	1 (10)		0 (0)
		Fall incident	0 (0)	0 (0)	0 (0)		0 (0)
		Wound infection	0 (0)	0 (0)	0 (0)		0 (0)
		Reoperation	0 (0)	0 (0)	0 (0)		0 (0)
		Others	8 (20)	8 (20)	3 (30)		0 (0)
**Hospital discharge variables**							
	**FAC^g^ at hospital discharge^a^, n (%)**						
		0-No functional mobility	0 (0)	5 (13)	0 (0)		1 (10)
		1-Dependent in mobility level 2	1 (3)	4 (10)	0 (0)		4 (40)
		2-Dependent in mobility level 1	9 (23)	17 (43)	0 (0)		2 (20)
		3-Independent mobility under supervision	22 (55)	9 (23)	8 (80)		1 (10)
		4-Independent mobility on a flat surface	8 (20)	5 (13)	2 (20)		2 (20)
	**FMS^h^ at hospital discharge, n (%)**						
		1-Freely mobile without aids	0 (0)	0 (0)	0 (0)		0 (0)
		2-Mobile outdoors with one aid	0 (0)	0 (0)	0 (0)		0 (0)
		3-Mobile outdoors with 2 aids or frame	3 (8)	2 (5)	1 (10)		0 (0)
		4-Some indoor mobility but never goes outside without help	37 (93)	33 (83)	9 (90)		9 (90)
		5-No functional mobility	0 (0)	5 (13)	0 (0)		1 (10)
	Katz-ADL6 at hospital discharge^a^, median (IQR)		4 (4-4)	4 (4-5)	4.5 (4-5)		4 (4-5)
	**Rehabilitation department skilled nursing home,** **n** **(%)**						
		Skilled nursing home A	22 (55)	23 (58)	9 (90)		5 (50)
		Skilled nursing home B	12 (30)	13 (33)	1 (10)		3 (30)
		Skilled nursing home C	6 (15)	4 (10)	0 (0)		2 (20)
	Length of hospital stay in days^a^, median (IQR)		7 (6-9)	8 (6.5-9)	6.5 (6-8)		7 (6-8)
**Geriatric rehabilitation variables**							
	MoCA^a,i^, median (IQR)		21.5 (18-24)	19.5 (17-22.5)	21 (20-25)		23 (18-26)

^a^Patient characteristics included in the ML models.

^b^PFMS: Prefracture Mobility Score.

^c^Katz-ADL6: Katz Index of Independence in Activities of Daily Living-6.

^d^CCI: Charlson Comorbidity Index.

^e^ASA: American Society of Anesthesiologists physical status classification.

^f^SNAQ: Short Nutritional Assessment Questionnaire.

^g^FAC: Functional Ambulation Categories.

^h^FMS: Fracture Mobility Score.

^i^MoCA: Montreal Cognitive Assessment.

### Feature Reduction

After applying PC analysis for the feature reduction of the features extracted from the physical activity data, 79 PCs were obtained. The graph of cumulative explained variance versus the number of PCs shows that 19 PCs explained 95% of the variance (Multimedia Appendix 2; Figure S1). These 19 PCs were included in the ML models.

In-hospital fall incidents, in-hospital wound infections, in-hospital reoperations, and other in-hospital complications were excluded because none of these complications occurred or the distributions were identical between the 2 lengths of stay groups. Hip fracture type was excluded from the ML models because it is strongly associated with surgical treatment [13]. Surgical treatment was regarded to be more patient-specific, as it depends on both fracture type and patient condition. FMS at hospital discharge was also excluded due to its strong association with functional ambulation category (FAC) at hospital discharge [13]. The FAC was considered more relevant because it is more widely used in geriatric rehabilitation to assess patient progress. Similarly, admission to which skilled nursing home was excluded due to its association with FAC at hospital discharge [13]. Notably, skilled nursing home A had more patients with lower FAC scores at hospital discharge compared to B and C. This was a coincidence, as the skilled nursing home placement procedure does not depend on the FAC. The FAC at hospital discharge was prioritized for its greater clinical relevance.

### ML Models

Based on the AUC of each ML model after the first 30 iterations (Multimedia Appendix 2; Figure S2), the SVM, NN, and EoDT ML models were selected for continuous Bayesian optimization.

The evaluation of the final hyperparameters after Bayesian optimization resulted in an NN consisting of only one layer with 2 neurons. Since this architecture was considered too simple and likely insufficient for capturing the complexity of the data, a new NN with 2 layers containing 102 and 51 neurons, respectively, was trained for further evaluation.

**Figure 4:**
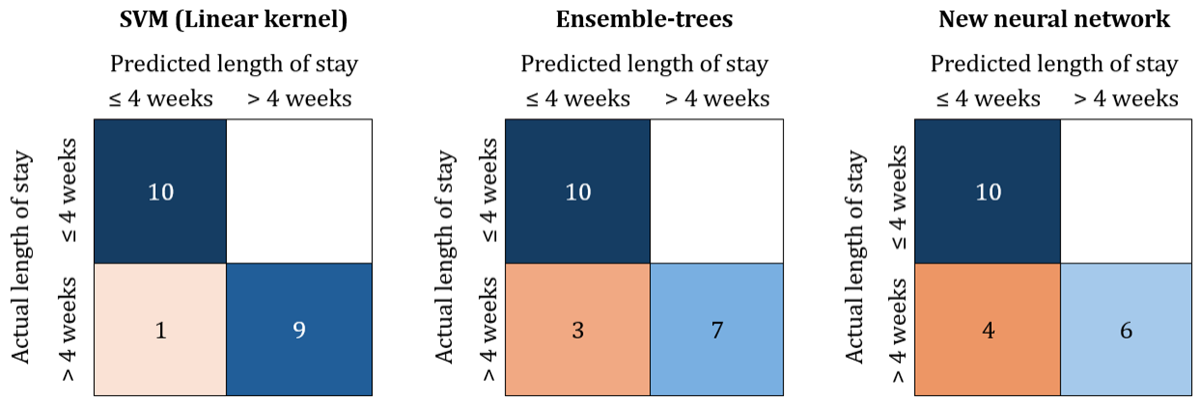
Confusion matrices of the final best-performing machine learning (ML) models (support vector machine [SVM], ensemble of decision trees [EoDT], and neural network [NN]) tested on the test set (n=20).

Figure 4 presents the confusion matrices of the final best-performing configuration of each remaining ML model type (SVM, EoDT, and NN) for the test set. The confusion matrix evaluates the performance of the ML models by comparing the actual length of stay with the predicted length of stay generated by the models. This provides an overview of the number of correctly classified and misclassified patients.

The performance of the final best-performing ML models, along with the actual length of stay for patients misclassified as ≤4 weeks, is presented in Table 2. The overall best results were achieved using the SVM model with a linear kernel (accuracy=0.95, 95% CI 0.85-1.00; precision=0.91, 95% CI 0.71-1.00; recall=1.00, 95% CI 1.00-1.00; *F*_1_-score=0.95238, 95% CI 0.83-1.00; AUC=0.97, 95% CI 0.83-1.00). The misclassified ≤4 weeks group included one patient with an actual length of stay of 42 days. The EoDT misclassified 3 patients with lengths of stay of 29, 29, and 42 days, while the new NN misclassified 4 patients with lengths of stay of 29, 30, 41, and 42 days.

**Table 2:** Evaluation of final best-performing machine learning (ML) models (SVM^a^, EoDT^b^, and neural network).

Machine learning models	Accuracy (95% CI)	Precision (95% CI)	Recall (95% CI)	*F*_1_-score (95% CI)	AUC^c^ (95% CI)	Actual LOS^d^ misclassified ≤4 weeks patients
SVM (linear kernel)	0.95 (0.85-1.00)	0.91 (0.71-1.00)	1.00 (1.00-1.00)	0.95 (0.83-1.00)	0.97 (0.88-1.00)	42 days
Ensemble-trees	0.85 (0.70-1.00)	0.77 (0.50-1.00)	1.00 (1.00-1.00)	0.87 (0.67-1.00)	0.97 (0.88-1.00)	29, 29, and 42 days
New neural network	0.80 (0.60-0.95)	0.71 (0.47-0.93)	1.00 (1.00-1.00)	0.83 (0.64-0.97)	0.92 (0.78-1.00)	29, 30, 41, and 42 days

^a^SVM: support vector machine.

^b^EoDT: ensemble of decision trees.

^c^AUC: area under the curve.

^d^LOS: length of stay.

**Figure 5:**
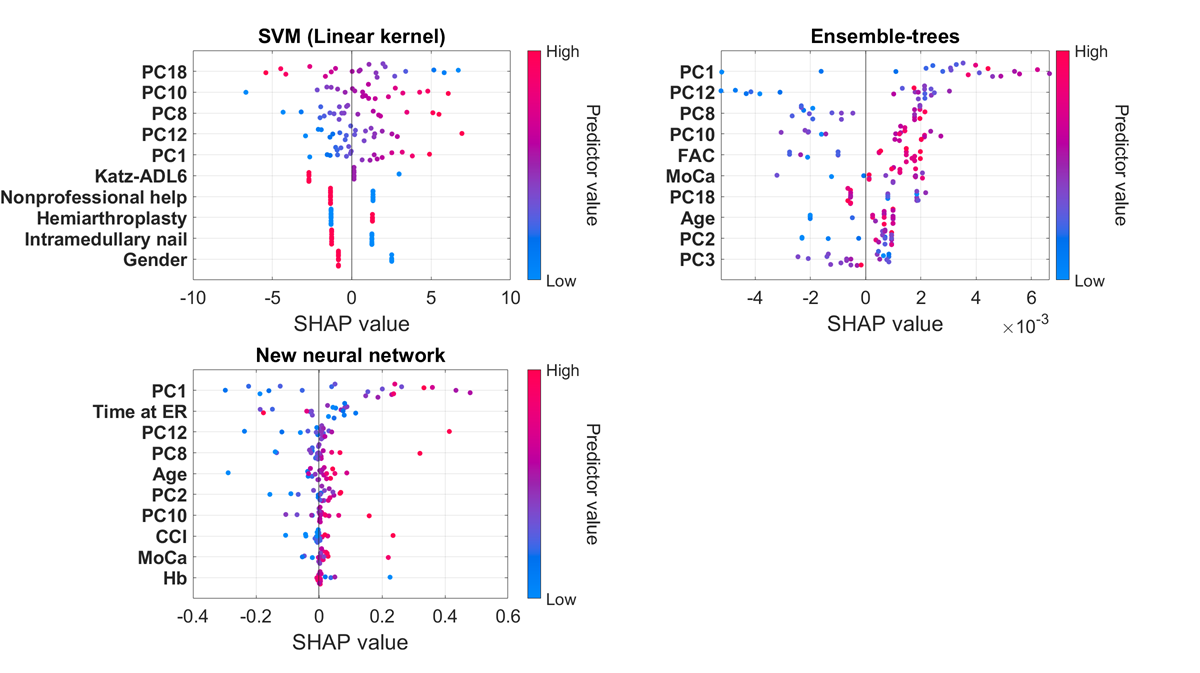
Shapley Additive Interpretation (SHAP) summary plots: the 10 most important features ranked from most to least impactful and their influence on predictions.

The 10 most important features of the final best-performing ML models, ranked from most to least impactful, and their influence on predictions can be seen in the results of the SHAP analysis (Figure 5). Each dot represents a patient from the test set, with its color indicating the actual feature value (predictor value: red for high values and blue for low values). The SHAP value of each dot represents the contribution of that feature to the prediction for an individual patient. It indicates how much the feature pushes the prediction toward one length of stay group or the other. Negative SHAP values push the prediction toward a length of stay of ≤4 weeks, while positive SHAP values push it toward a length of stay of >4 weeks.

For example, in the Shapley summary plot of the SVM (linear kernel) model, the feature availability of nonprofessional help appears blue on the right. This indicates that patients with no availability of nonprofessional help (0=no availability, 1=availability) have positive SHAP values, pushing predictions toward a length of stay of >4 weeks. Based on the SHAP analysis, it can be seen that the most important features for predicting the length of geriatric rehabilitation stay, across the 3 best-performing ML models, were the PCs (weighted combinations of features extracted from the physical activity data); time in the emergency room (ER); FAC at hospital discharge; age; Katz Index of Independence in Activities of Daily Living-6 (Katz-ADL6) at hospital discharge; MoCA; availability of nonprofessional help; surgery type; Charlson Comorbidity Index (CCI); gender; and hemoglobin level at hospital admission.

In the ablation study, the best-performing ML model trained on physical activity data only outperformed the model trained on patient characteristics only. The combined dataset yielded the highest predictive performance. The confusion matrices and detailed performance metrics for the ablation study are provided in Multimedia Appendix 3.

## Discussion

### Principal Findings

This study developed ML models for the early prediction of a geriatric rehabilitation stay of ≤4 weeks in older patients after hip fracture surgery, using continuously monitored physical activity data and patient characteristics. This resulted in several ML models with varying overall predictive performance. The SVM was the best-performing model, demonstrating excellent predictive capability with an accuracy of 0.95 (95% CI 0.85-1.00), precision of 0.91 (95% CI 0.71-1.00), recall of 1.00 (95% CI 1.00-1.00), *F*_1_-score of 0.95 (95% CI 0.83-1.00), and AUC of 0.97 (95% CI 0.88-1.00). These initial results suggest that early prediction of a geriatric rehabilitation stay of ≤4 weeks is promising.

In our previous study, the FAC at hospital discharge, premorbid living situation, postoperative weight-bearing protocol, surgery type, in-hospital delirium, and in-hospital heart failure were identified as key factors affecting the length of geriatric rehabilitation stay [13]. Among all these previously identified predictors, the current study confirmed that FAC at hospital discharge and surgery type are key features for predicting a geriatric rehabilitation stay of ≤4 weeks. Postoperative weight-bearing protocol, in-hospital delirium, and in-hospital heart failure did not emerge as key predictors in the best-performing models. A possible explanation is that these factors occurred relatively infrequently in our study population, limiting their predictive power in this context. Furthermore, whereas the previous study focused solely on identifying predictors for the length of geriatric rehabilitation stay, the current study developed and evaluated actual prediction models, offering potential for early discharge planning.

A substantial amount of other research also exists on the factors influencing the length of stay after hip fracture surgery. However, many of these studies focus on factors influencing the length of hospital stay [28,30], or the length of stay in other rehabilitation settings (eg, private care rehabilitation, in-hospital rehabilitation, or both the acute hospital and rehabilitation phases) [21-24], making them difficult to compare with this study. These previously identified predictors for the length of geriatric rehabilitation stay include age [22-24]; fracture type [22]; American Society of Anesthesiologists physical status classification score [20,21]; complications (wound infection, delirium, urinary tract infection, and pneumonia) [22,23]; comorbidities (Parkinson disease, diabetes, and dementia) [22-24]; gait status at hospital discharge [20]; living situation prior to the hip fracture (alone or together) [24,25]; use of mobility aids prior to the hip fracture [21,25]; Barthel score at geriatric rehabilitation admission [25]; Functional Independence Measure score at rehabilitation admission [23]; Abbreviated Mental Test Score (AMTS) [21]; and pain score at rehabilitation admission [24]. Among these previously identified predictors, this study further confirmed that age, comorbidities (CCI), gait status at hospital discharge (FAC), an assessment tool to evaluate a patient’s functional ability to perform activities of daily living (Barthel score or Katz-ADL6), and a cognitive score (AMTS or MoCA) are key features for predicting a geriatric rehabilitation stay of ≤4 weeks.

Unlike previous studies, which did not incorporate continuously measured physical activity, this study included this aspect, providing a more detailed insight into the effect of physical activity on the length of geriatric rehabilitation stay. Consequently, the PCs (weighted combinations of features extracted from the physical activity data) of this study emerged as one of the most important features for predicting a geriatric rehabilitation stay of ≤4 weeks. The findings of this study underscore the critical role of physical activity and align with previous studies emphasizing the positive impact of early mobilization and physical activity on the functional recovery of hip fracture patients [14-16,34]. Recent research further supports this by demonstrating that higher levels of physical activity at the time of rehabilitation admission are associated with greater independence in activities of daily living at discharge [35]. Early mobilization may help maintain postural muscle function, leading to improved functional recovery [35]. In contrast, prolonged bed rest, associated with low levels of physical activity, has been linked to a decline in physical function and reduced independence in activities of daily living due to decreased oxygen consumption, metabolic changes, and muscle atrophy [36,37]. Patients with lower physical activity levels at rehabilitation admission may experience slower functional recovery, prolonged dependence in activities of daily living, and consequently a longer geriatric rehabilitation stay.

In addition to physical activity, this study identified other important predictors of the length of geriatric rehabilitation stay that have not been examined before in previous research. Specifically, the availability of nonprofessional help, time in the ER, and hemoglobin level at hospital admission emerged as important predictors. The availability of nonprofessional help was included as it represents a key aspect of functional social support (social relationships that fulfill particular functions in times of need), which has been shown to be associated with rehabilitation outcomes in older adults with hip fractures [27]. Time spent in the ER was included, as recent research has found an association with hospital length of stay [30], although the reason for this association remains unclear. Hemoglobin level at hospital admission was also included, as recent research has found an association with hospital length of stay due to delayed recovery and increased complication risk in patients with low hemoglobin levels [28].

The remaining most important feature across all ML models, gender, also does not align with previously identified predictors of the length of geriatric rehabilitation stay. However, gender has been included as a variable in earlier studies, although it was not found to be a significant predictor. A possible explanation for its role as a key feature in this study, but not in previous ones, may lie in the use of different analytical techniques. In this study, we used ML techniques, whereas previous research relied on traditional statistical analyses.

Overall, while many of the key features for predicting a geriatric rehabilitation stay of ≤4 weeks identified in this study align with previous research, this study also uncovered other predictors, including weighted combinations of features extracted from continuously measured physical activity data, availability of nonprofessional help, and time spent in the ER. These findings highlight the potential influence of factors often overlooked in traditional statistical analyses.

When examining the misclassified ≤4 weeks patients of the 3 best-performing ML models in more detail, the EoDT has 2 patients with a length of stay of 29 days, and the new NN has one patient with a length of stay of 29 days and another with a length of stay of 30 days. However, misclassifying a 29- and 30-day stay as ≤4 weeks may not be a clinically significant issue, as it is close to 28 days. Furthermore, the new NN misclassified a patient with a length of stay of 41 days. However, this patient developed a complication during rehabilitation, which may have contributed to the incorrect prediction. Since complications occurring after the first seven days of geriatric rehabilitation are not taken into account in the models, this may explain the misclassification. In clinical practice, however, treatment plans are adjusted when complications arise, meaning that such a case would likely have been identified as requiring a longer stay regardless. The other 3 misclassified patients across the 3 best-performing ML models had a length of stay of 42 days. No clear explanation was found based on the available data.

### Strengths and Limitations of This Study

To the best of our knowledge, this is the first study to develop and compare ML models for predicting a geriatric rehabilitation stay of ≤4 weeks in a skilled nursing home at an early rehabilitation stage, using a novel experimental approach with continuously monitored physical activity data and patient characteristics in older patients after hip fracture surgery. Another strength of our study is the inclusion of a diverse range of features from both the physical and social domains, with particular emphasis on the integration of continuously monitored physical activity data. In addition, the MOX1 accelerometer was well tolerated by patients and was easily integrated into the clinical workflow, requiring minimal effort from clinical staff and demonstrating the feasibility of continuous activity monitoring in this population.

Regarding the limitations, a complete dataset was required for ML model development, leading to the exclusion of many patients due to missing a single feature. No data imputation was applied in this study, as the aim was to avoid introducing potential bias from imputed values. This requirement for complete data reduced the sample size. A practical limitation contributing to missing physical activity data was the inability to check the battery level of the MOX1 accelerometer during use. Although the lights of the accelerometer show whether it is actively measuring, clinical staff did not routinely check this, as the wearable required no daily attention. As a result, wearables occasionally ran out of battery unnoticed, leading to the loss of multiple days of activity data. While model performance was already high, having complete data for all patients could have potentially increased the reliability and robustness of the analyses. Second, given the focus on ML model development, the sample size may be relatively small, making some models prone to overfitting. To mitigate this risk, techniques such as cross-validation and feature reduction were applied. Third, although length of geriatric rehabilitation stay is a continuous variable, this study formulated the problem as a classification task (≤4 weeks vs >4 weeks). This cutoff reflects a clinically meaningful decision point with substantial impact on early discharge planning. For clinicians, knowing after one week whether a patient is likely to be discharged within 4 weeks is far more actionable than attempting to predict the exact number of days. Predicting the exact length of stay in days would introduce unnecessary complexity, require substantially more data, and is not essential for guiding early clinical decisions. Therefore, we consider the current classification framework to be the most suitable and clinically relevant approach for guiding early discharge planning. Future research is recommended to explore appropriate data imputation strategies in a cautious manner, to reduce data loss while ensuring that bias is not introduced. For categorical patient characteristics such as the availability of nonprofessional help, imputation may be challenging and not always be desirable, highlighting the need for careful consideration of which variables to impute. In addition, future work is recommended to validate the models with a larger patient population, refine them if necessary, and integrate the best-performing model into clinical practice.

### Clinical Interpretation

The results of this study indicate that early prediction of a geriatric rehabilitation stay of ≤4 weeks or less in a skilled nursing home for older patients after hip fracture surgery is feasible. This marks the first step toward a more future-proof geriatric rehabilitation system. Identifying patients with an expected stay of ≤4 weeks or less after 7 days of rehabilitation enables timely discharge planning and coordination of support at home. Such planning can smooth the transition toward home and prevent discharge delays, which is important to ensure optimal bed occupancy.

### Conclusion

Several ML models were developed to predict a geriatric rehabilitation stay of ≤4 weeks in a skilled nursing home for older patients after hip fracture surgery, using continuously monitored physical activity data from the first week of geriatric rehabilitation and patient characteristics. Among these models, the SVM proved to be highly accurate in its predictions with an accuracy of 0.95 (95% CI 0.85-1.00), precision of 0.91 (95% CI 0.71-1.00), recall of 1.00 (95% CI 1.00-1.00), *F*_1_-score of 0.95 (95% CI 0.83-1.00), and AUC of 0.97 (95% CI 0.88-1.00).

## Appendix

Multimedia Appendix 1 Detailed explanation of the extracted continuously monitored physical activity features.

Multimedia Appendix 2 Feature reduction and machine learning model development.

Multimedia Appendix 3 Confusion matrices and detailed performance metrics for the ablation study.

## Abbreviations

AMTS: Abbreviated Mental Test Score
ASA: American Society of Anesthesiologists physical status classification
AUC: area under the curve
CCI: Charlson Comorbidity Index
EoDT: Ensemble of decision trees
ER: emergency room
FAC: functional ambulation categories
Katz-ADL6: Katz Index of Independence in Activities of Daily Living-6
ML: machine learning
MoCA: Montreal Cognitive Assessment
NB: naïve Bayes
NN: neural network
PFMS: Prefracture Mobility Score
PC: principal component
SHAP: Shapley Additive Interpretation
SVM: support vector machine
ZGT: Ziekenhuisgroep Twente

## Data Availability

The datasets analyzed during this study are not publicly available due to the fact that our patients did not give permission to share the data publicly but are available from the corresponding author on reasonable request.
