# Supplementary material for: Predicting Geriatric Rehabilitation Stays of ≤4 Weeks After Hip Fracture Surgery: Machine Learning Approach Using Physical Activity and Patient Data
Source: JMIR Rehabil Assist Technol. 2026 Feb 23;13:e79331. doi: 10.2196/79331 (PMC12972686; doi:10.2196/79331)
Supplement: Multimedia Appendix 1 [file rehab_v13i1e79331_app1.docx]

**Appendix 1. Detailed explanation of the extracted continuously monitored physical activity features**

| **Feature** | **Explanation** |
| --- | --- |
| Overall mean | The overall mean (equation 1) was calculated to characterize the average value of each metric:  $Mean=\bar{x}=\frac{1}{n}\sum_{i=1}^{n} x_{i} \left( 1 \right)$  where $x_{i}$ is the $i^{th}$ metric value in a window of $n$ data points. |
| Overall standard deviation (SD) | The overall standard deviation (equation 2) was calculated to characterize the variability of each metric and was defined as the square root of the variance:  $SD=\sqrt{\frac{\sum_{i=1}^{n} \left( x_{i}- \bar{x} \right)^{2}}{n}} (2)$  where $x_{i}$ is the $i^{th}$ metric value in a window of $n$ data points, and $\bar{x}$ the mean metric value within that window. |
| Weekend mean | The weekend mean was calculated like equation 1 to characterize the average value of each metric at the weekend: |
| Weekend SD | The weekend standard deviation was calculated like equation 2 to characterize the variability of each metric at the weekend and was defined as the square root of the variance: |
| Weekday mean | The weekday mean was calculated like equation 1 to characterize the average value of each metric at the weekdays: |
| Weekday SD | The weekend standard deviation was calculated like equation 2 to characterize the variability of each metric at the weekdays and was defined as the square root of the variance: |
| Median | The median was calculated to characterize the “middle value” of each metric. |
| Interquartile range (IQR) | The IQR was calculated to characterize the statistical dispersion of each metric and was defined as the difference between the 75th and the 25th percentile of the values of each metric. |
| Root mean square (RMS) | The RMS (equation 3) was used to characterize the quadratic mean of each metric and was defined as the square root of the mean square:  $RMS=\sqrt{\frac{x_{1}^{2}+x_{2}^{2}+\ldots+x_{n}^{2}}{n}} (3)$  where $x$ is the overall metric value and $n$ the number of data points within a window. |
| Variance | The variance (equation 4) was used to quantify the spread of each metric around its mean, providing insight into the variability of the data:  $Variance=\frac{\sum_{i=1}^{n} {(x_{i}-\bar{x})}^{2}}{n} (4)$  where $x_{i}$ is the $i^{th}$ metric value in a window of $n$ data points, and $\bar{x}$ the mean metric value within that window. |
| Minimum value | The minimum value of each metric. |
| Maximum value | The maximum value of each metric. |
| Minimum-maximum range | The minimum-maximum range was calculated to characterize a patients’ range of each metric. |
| Coefficient of variance (CV) | The CV (equation 5)was used to express the relative variability of each metric, normalizing the standard deviation by the mean:  $CV=\frac{SD}{\bar{x}}\times100\% \left( 5 \right)$  where $SD$ is the standard deviation of the metric value in a window of $n$ data points, and $\bar{x}$ the mean metric value within that window. |
| Skewness | The skewness (equation 6) was used to measure the asymmetry of the distribution of each metric:  $Skewness=\frac{1}{n}\sum_{i=1}^{n} \left( \frac{x_{i}-\bar{x}}{SD} \right)^{3} (6)$  where $x_{i}$ is the $i^{th}$ metric value in a window of $n$ data points, $SD$ is the standard deviation and $\bar{x}$ the mean metric value within that window. |
| Kurtosis | The kurtosis (equation 7) was calculated to quantify the tailedness of the distribution of each metric:  $Kurtosis=\frac{1}{n}\sum_{i=1}^{n} \left( \frac{x_{i}-\bar{x}}{SD} \right)^{4} (7)$  where $x_{i}$ is the $i^{th}$ metric value in a window of $n$ data points, $SD$ is the standard deviation and $\bar{x}$ the mean metric value within that window. |
| Coefficient a, b, c | Mathematical coefficients describing a third-degree polynomial curve (coefficient a, b, and c) were calculated to characterize the shape of the pattern of each metric over time. All coefficients were extracted by fitting each metric curve with a third-degree polynomial curve fit. Coefficient a, b, and c of the third-degree polynomial equation $y=ax^{3}+bx^{2}+cx+d$ obtained from the third-degree polynomial curve fit were defined as coefficient a, b, and c, respectively. |
