# Supplementary material for: Predicting Geriatric Rehabilitation Stays of ≤4 Weeks After Hip Fracture Surgery: Machine Learning Approach Using Physical Activity and Patient Data
Source: JMIR Rehabil Assist Technol. 2026 Feb 23;13:e79331. doi: 10.2196/79331 (PMC12972686; doi:10.2196/79331)
Supplement: Multimedia Appendix 2 [file rehab_v13i1e79331_app2.docx]

**Appendix 2. Feature reduction and machine learning model development**

| Figure 1. Explained variance of principal components. |
| --- |
| 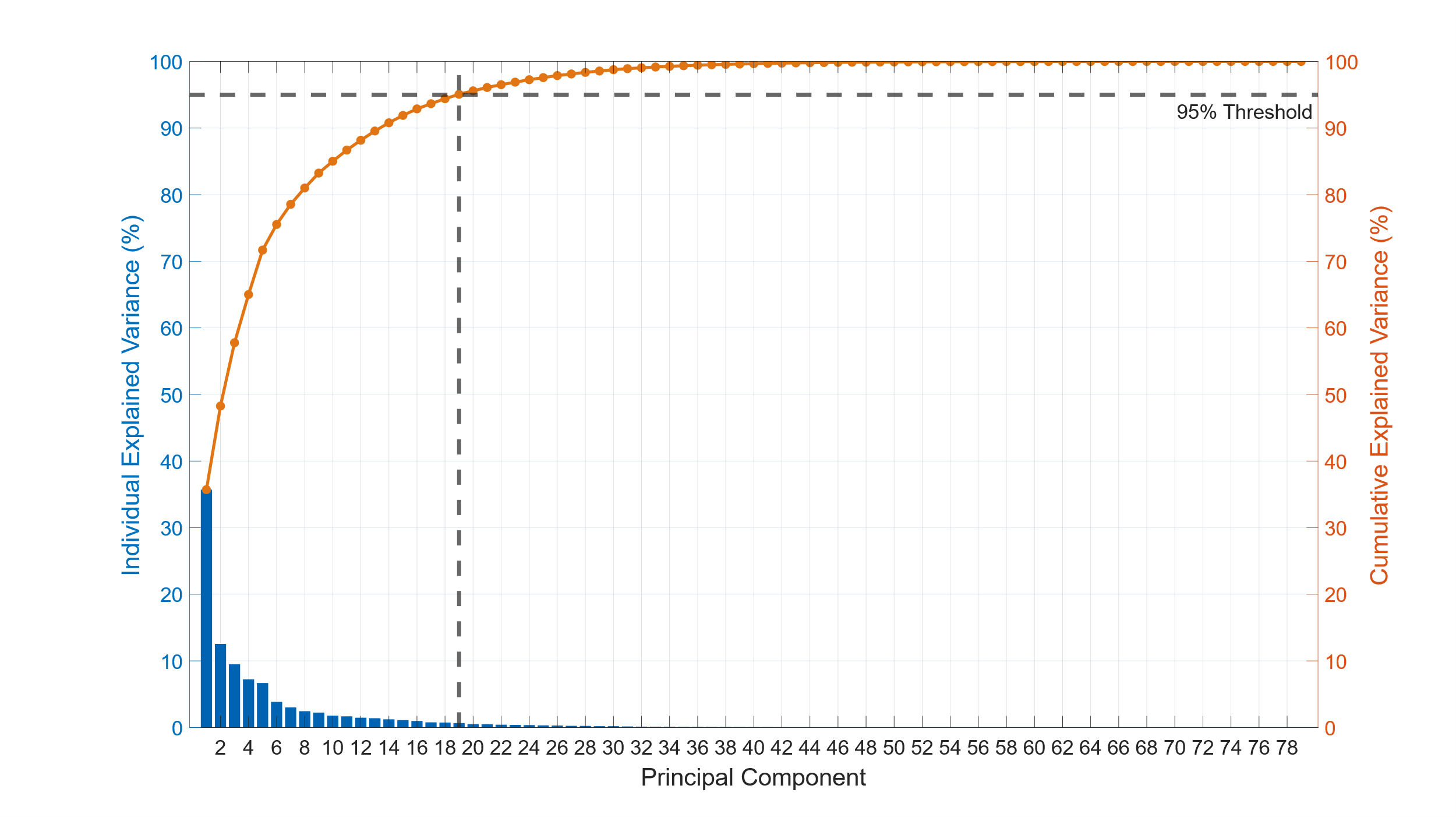 |

| Figure 2. Area under the receiver operating characteristics curve. |
| --- |
| 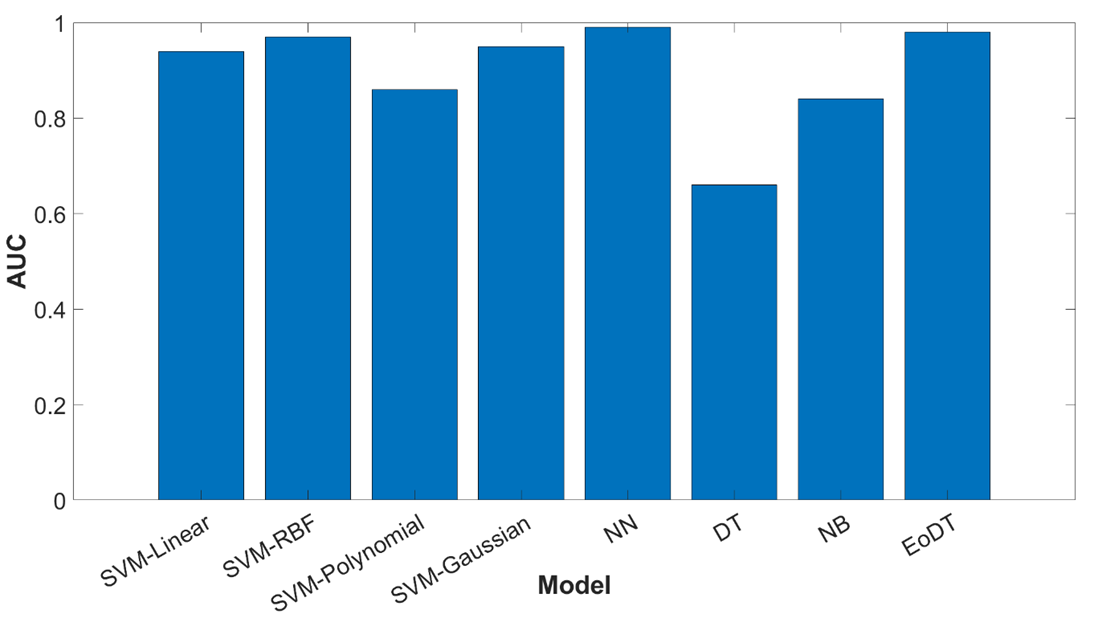 |
