# Supplementary material for: Predicting Geriatric Rehabilitation Stays of ≤4 Weeks After Hip Fracture Surgery: Machine Learning Approach Using Physical Activity and Patient Data
Source: JMIR Rehabil Assist Technol. 2026 Feb 23;13:e79331. doi: 10.2196/79331 (PMC12972686; doi:10.2196/79331)
Supplement: Multimedia Appendix 3 [file rehab_v13i1e79331_app3.docx]

**Appendix 3. Confusion matrices and detailed performance metrics for the ablation study**

| Figure 1. Confusion matrices from the ablation study of the best-performing SVM (linear kernel) on test set (*n* = 20). |
| --- |
| 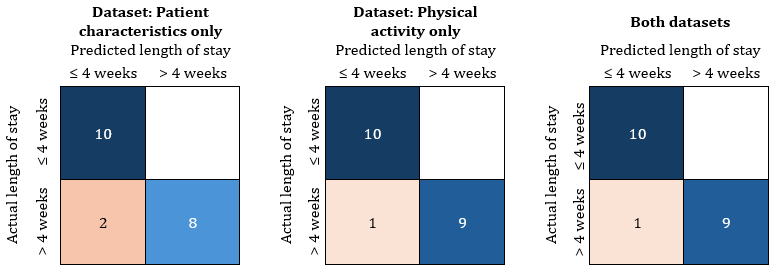 |

| Table 1. Performance metrics of the best-performing SVM (Linear kernel) across different dataset configuration in the ablation study. | | | | | |
| --- | --- | --- | --- | --- | --- |
|  | Accuracy | Precision | Recall | F_1_-score | AUC |
| Dataset: Patient characteristics only | 0.90  [0.75, 1.00] | 0.83  [0.58, 1.00] | 1.00  [1.00, 1.00] | 0.91  [0.74, 1.00] | 1.00  [1.00, 1.00] |
| Dataset: Physical activity only | 0.95  [0.85, 1.00] | 0.91  [0.70, 1.00] | 1.00  [1.00, 1.00] | 0.95  [0.82, 1.00] | 0.95  [0.82, 1.00] |
| Both datasets | 0.95  [0.85, 1.00] | 0.91  [0.71, 1.00] | 1.00  [1.00, 1.00] | 0.95  [0.83, 1.00] | 0.97  [0.88, 1.00] |
